# Cross-species comparison of airway epithelium transcriptomics

**DOI:** 10.1016/j.heliyon.2024.e38259

**Published:** 2024-09-22

**Authors:** Biyu Gui, Qi Wang, Jianhai Wang, Xue Li, Qi Wu, Huaiyong Chen

**Affiliations:** a Department of Respiratory Medicine, Haihe Clinical School, Tianjin Medical University, Tianjin, 300350, China; b Department of Basic Medicine, Haihe Hospital, Tianjin University, Tianjin, 300350, China; c Department of Stomatology, Haihe Hospital, Tianjin University, Tianjin, 300350, China; d Tianjin Institute of Respiratory Diseases, 300350, Tianjin, China; e Tianjin Key Laboratory of Lung Regenerative Medicine, Haihe Hospital, Tianjin University, Tianjin, 300350, China

**Keywords:** Cross-species comparison, Lung, Asthma, scRNA-seq

## Abstract

Studies of lung transcriptomics across species are essential for understanding the complex biology and disease mechanisms of this vital organ. Single-cell RNA sequencing (scRNA-seq) has emerged as a key tool for understanding cell dynamics across various species. However, comprehensive cross-species comparisons are limited. Therefore, the aims of this study was to investigate the transcriptomic similarities and differences in lung cells across four species—humans, monkeys, mice, and rats—in healthy and asthma conditions using scRNA-seq. The results revealed significant transcriptomic similarities between monkeys and humans and significant cross-species conservation of cell-specific marker genes, transcription factors (TFs), and biological pathways. Additionally, we explored sex differences, identifying distinct sex-specific expression patterns that may influence disease susceptibility. These insights refine our understanding of the mechanism underlying airway cell biology across species and have important implications for studying lung diseases, particularly the mechanisms of mucus clearance in asthma.

## 1 Introduction

The mammalian lung is a highly complex organ crucial for respiratory function and immune defense. The human lung contains between 40 and 61 distinct cell types [1,2], highlighting the challenges in understanding respiratory diseases such as asthma. Mice, rats and monkeys are commonly used as model organisms for studying human lung biology. Comparative analyses of lung cells across species can reveal conserved processes and species-specific characteristics, providing valuable insights into the selection of appropriate models and identification putative zoonotic reservoirs [3,4]. However, comprehensive studies of lung transcriptomics across species remain limited, impeding progress in this field.

Single-cell RNA sequencing (scRNA-seq) offers unprecedented resolution for examining cellular heterogeneity and dynamic responses of lung cells under various health conditions. This technology facilitates detailed characterization of cell types within tissues, yielding deep insights into their functions and interactions within their native environments [2,5,6]. The largest human lung single-cell transcriptomics study, the *Human Lung Cell Atlas*, has shown that sex is associated with transcriptomic variation [2]. Despite significant advances in scRNA-seq and its application in sex-based comparisons, cross-species comparisons of healthy and diseased lung tissues at the single-cell level remain insufficient.

Asthma, a common chronic inflammatory respiratory disease, is characterized by the presence of airway mucus plugs [7]. However, animal models that accurately mimic airway mucus plugging are lacking. This phenomenon is primarily associated with epithelial cells, particularly with the secretion of highly viscous mucin 5AC (MUC5AC) mucus by mucus-secreting cells in response to interleukin 13, which also damages ciliated cells and impairs mucus clearance [8,9]. Club cells are the primary MUC5AC-secreting cells [10]. The airways are lined with a substantial number of club and ciliated cells, which, along with the epithelial cells of the alveoli, form a critical barrier. Our previous research demonstrated MUC5AC mucus metaplasia in club cells and its relationship with OVA-induced allergic airway inflammation [11]. Comparing transcriptomic similarities between club and ciliated cells across species in asthma models is crucial for elucidating the mechanism underlying airway mucus plugging in animal models.

The aim of this study aims was to address these knowledge gaps by analyzing the lungs of four species—humans, monkeys, mice, and rats—using scRNA-seq. We focused on identifying common and unique transcriptomic features, examining the effect of sex on gene expression, and evaluating the reliability of animal models in simulating human asthma. The goal was to provide a detailed account of airway epithelial cells across various species and sexes, offering a valuable reference for future research by integrating comprehensive bioinformatics tools and advanced statistical techniques.

## 2 Methods

### 2.1 Samples and datasets

Lung single-cell data from multiple species were retrieved from the GEO database and included data from healthy human lung cells obtained from disease-free lung tissue and lung tissues from other species, particularly mice, which were maintained under standard conditions (normal diet, normal atmospheric pressure, and oxygen levels) and treated with balanced salt solutions in certain cases. Monocyte transcriptome data were excluded, additionally, samples sorted through fluorescence-activated cell sorting and selected using the kit were excluded. Furthermore, a Pubmed literature search was conducted to supplement the sample size. Healthy human specimens were restricted to donors under 50 years of age for consistency, aligning with the euthanizing age of the animal. Owing to specimen scarcity, the asthma model dataset included only male specimens up to 65 years of age.

### 2.2 Data processing

Analyses were performed using R software (version 4.2.1) [12]; the codes are provided in supplementary materials. Single-cell data were processed using Seurat (version 4.4.0) [13], while other file reading and data processing tasks were handled using the tidyverse package (version 1.3.2) [14]. Lung samples was subjected to initial processing to eliminate doublets using DoubletFinder (version 2.0.4) [15] and Matrix package (version 1.6–5) [16] and to remove low-quality cells with Seurat. Initial filtering criteria included a minimum of 200 unique molecular identifier per cell, a minimum of three cells per gene, and no more than 10–30 % mitochondrial gene expression. Samples were merged for a standardized workflow, including normalization, scaling, and clustering using Seurat, with batch effects managed using the Harmony package (version 1.2.0) [17]. The cells were primarily classified into six main clusters and further refined into fine cell types using canonical markers. This procedure was replicated for the asthma dataset, with the aim of analyzing common cell types across species.

### 2.3 Orthologous gene symbol conversion

Gene symbols from the three additional species were converted to their corresponding human gene symbols using orthologous gene lists from the BioMart database (https://www.ensembl.org/biomart/martview) to facilitate the comparison of lung cell similarities and differences between humans and other species. Direct conversion was applied for a one-to-one match between a nonhuman and human gene symbol. When multiple nonhuman gene symbols corresponded to a single human gene symbol, the nonhuman gene expression levels within each cell were aggregated before conversion. Conversely, if a single nonhuman gene symbol corresponded to multiple human gene symbols, the nonhuman gene expression level was duplicated for each corresponding human gene symbol during conversion.

### 2.4 Sampled data

A random seed was set, and a certain number of shared cells were randomly selected from each of the four species. If the number of available cells for a particular species was insufficient, the sampling size was adjusted to the minimum number of cells available among the four species.

### 2.5 Geneset score

Fifty human hallmark gene sets [18], representing well-defined biological states or processes, were retrieved from the Molecular Signatures Database (MsigDB, http://www.gsea-msigdb.org/gsea/msigdb) using the msigdbr package (version 7.5.1) [18]. Asthma and ciliated cell-associated gene sets (systematic name: M35640, M13950, M38065, and M48824) were also downloaded from the molecular signature database (MsigDB), with orthologous gene symbol conversion applied for data preparation. The efficacy of these gene sets in the down-sampled gene × cell matrices was assessed using the AddModuleScore function in the Seurat package.

### 2.6 Cell-type marker genes and differentially expressed genes

Cell-type marker genes were analyzed across all cells using the FindAllMarkers function of the Seurat package. Differentially expressed genes (DEGs) between healthy and asthma groups were analyzed using the FindMarkers function of the Seurat package. Genes were selected based on the following stringent criteria: |fold-change| > 1.5, adjusted *p* < 0.05, and percentage expression in >25 % of cells in at least one cell group.

### 2.7 Gene ontology and Kyoto encyclopedia of genes genomes enrichment

Symbol IDs were converted to Ensembl gene IDs using the bitr function of the clusterProfiler package (version 4.6.2) [19]. Species annotation libraries were sourced from the org.Hs.eg.db, org.Mmu.eg.db, org.Mm.eg.db, and org.Rn.eg.db packages (version 3.16.0) [[20], [21], [22], [23]]. Further analyses of gene ontology (GO) and Kyoto Encyclopedia of Genes and Genomes (KEGG) pathway enrichment were performed using the enricher function of the clusterProfiler package. Considering multiple comparisons, terms were filtered with a criterion of *q* < 0.05.

### 2.8 Transcription factors

TFs were analyzed using the SCENIC package (version 1.3.1) [24]. Owing to the limited availability of reference databases that include data on only humans and mice, the human (hg38) TF database (https://resources.aertslab.org/cistarget/databases) was employed to map the networks of TFs and their target genes across all cell-type marker genes in the human lung and their orthologs in other species. Initially, cell-type marker genes or DEGs for common cell types were extracted from the aggregate gene set, and cells from each dataset were randomly sampled as previously described. Nonhuman cell-type marker genes were aligned with their human orthologs, as outlined in section 2.3. These down-sampled count matrices were then used as input for mapping the coexpression regulons of TFs and their target genes using the GENIE3 package (version 1.20.0) [24,25]. To assess the correlations between TFs and their corresponding target genes for each regulon, the RcisTarget package (version 1.18.2) [24] was employed, retaining only targets that exhibited positive correlations. Based on gene expression levels, regulon activity within each cell was quantified using the AUCell package (version 1.20.2) [24]. Cross-species comparisons of regulon classifications and activities were subsequently performed using the “cor.test” function of stats packages (version 4.2.1) [12] to identify similarities and differences among species.

### 2.9 Statistical analysis

For continuous variables, normality was assessed using the Shapiro–Wilk test from the stats packages. Variables conforming to a normal distribution are described using mean ± standard deviation, whereas the non-conforming ones are described using median and quartiles, as shown in the violin plots. Correlation analysis for two paired continuous variables was performed using the cor.test function, with Pearson's test applied to normally distributed data and Spearman's test applied to non-normally distributed data. Additionally, the Mantel's test (“Mantel.rtest”) function from the ade4 package (version 1.7–22) [26] was utilized to assess the similarity between two expression matrices. A two-sided p-value <0.05 was considered statistically significant (∗*p* < 0.05, ∗∗*p* < 0.01, ∗∗∗*p* < 0.001).

### 2.10 Graphical representation

Uniform manifold approximation and projection (UMAP) plots were generated using the Seurat package. Chord diagrams were created using the Circlize package (version 0.4.16) [27], and heatmaps were produced using the ComplexHeatmap package (version 2.15.4) [28]. Venn diagrams were constructed using the VennDiagram packages (version 1.7.3) [29]. Most other graphics were created using the ggplot2 package (version 3.5.0) [30], which is part of the tidyverse package. Additional visualizations were facilitated by extensions of ggplot2, including the GGally package (version 2.2.1) [31], cowplot package (version 1.1.3) [32], and ggpubr package (version 0.6.0) [33].

## 3 Results

### 3.1 Lung transcriptome atlas across species

**Fig. 1:**
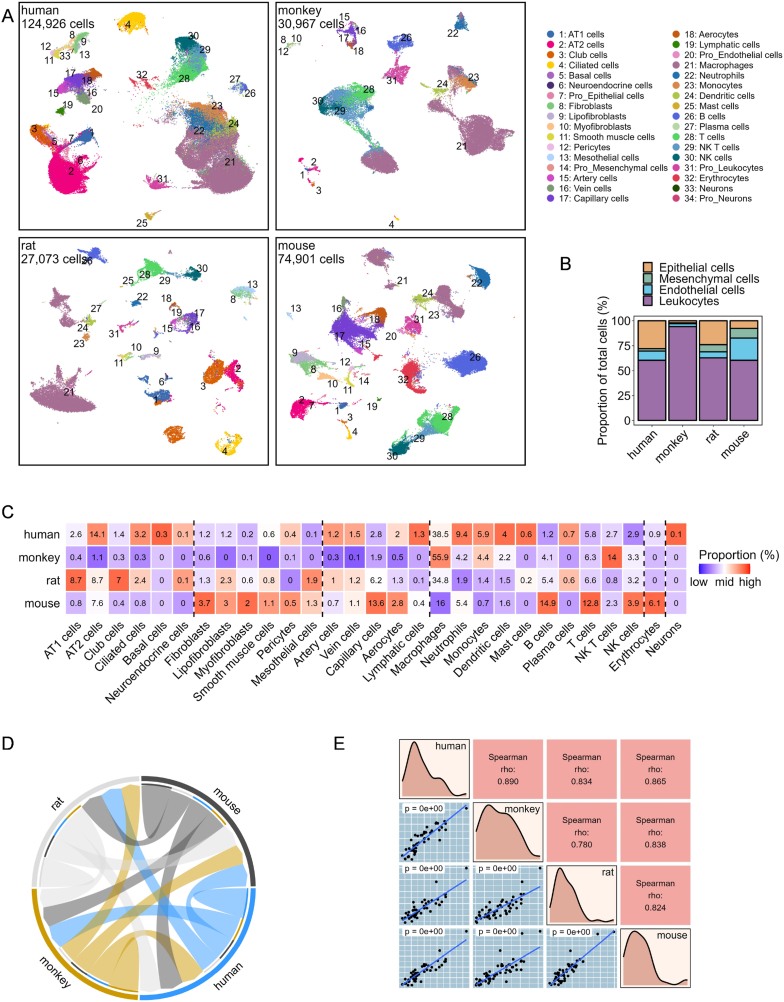
**Full-transcriptome landscape of healthy lung cells and cross-species comparisons.** (A) UMAP plots show the distribution of 34 fine cell types in the lungs of healthy humans, monkeys, rats, and mice, respectively. Cell types are color-coded and numbered, with the total number of cells for each dataset showing in the top-left corner of each plot. (B) Proportions of four main cell clusters across different species. (C) Proportions of 29 fine cell types across different species. Numbers within the image represent cell ratios, and the proportions for each specific cell type are normalized across species, with color-filled based on the normalized values. (D) Chord plot illustrates the correlation of full-transcriptome expression levels and their percentage in randomly sampled lung cells across species. Arrow width represents the correlation coefficient from Mantel's test between species pairs connected by a chord. (E) Correlation analysis of randomly sampled lung cells cross-species utilizing module scores for 50 hallmark gene sets from the MsigDb dataset. Each black dot in the bottom-left part represents a gene set, with its coordinates representing the module scores for the corresponding two species labeled diagonally. Blue trend lines are derived from a linear model. The correlation coefficient for each dot plot is shown in the corresponding position in the top-right corner, with the chosen calculation method depending on the distribution of the data. Diagonal plots represent the density distribution of the four species.

Lung samples from the four species were initially screened by donor age, excluding humans >50 years of age, resulting in 74 samples used for analysis (Fig. S4A). ScRNA-seq data from these samples were subjected to rigorous quality control, including the removal of doublets and low-quality cell. Subsequently, the data were merged into species-specific matrices. Following processing with the Seurat workflow, 257,867 cells were classified into 29 distinct cell types and 5 corresponding proliferative states utilizing canonical markers (Fig. 1A, Supplementary Table S1). While all 34 cell types were comprehensively mapped in the human dataset, fewer cells were identified in other species, particularly monkeys. This suggests a correlation between the number of cells and the precision of cell type annotation.

The proportions of each cell cluster within each species were calculated. Leukocytes dominated the cellular composition, representing more than 50 % of the total cells in all species except rats (Fig. 1B). Although the proportion of cell clusters usually depends on sample spots and digestive enzymes, the predominance of leukocytes in lung tissue proportion indicates a consistent pattern. Additionally, species differences were observed. For example, humans had higher proportions of alveolar epithelial and myeloid cells, whereas monkeys, rats, and mice exhibited higher counts of macrophages, airway epithelial and endothelial cells, and mesenchymal cells, respectively (Fig. 1C).

A full-transcriptome approach was employed to examine the similarities and differences among the datasets of common cell types for cross-species transcriptomic comparisons. This process involved the random sampling of 25 cells from each of the 18-cell types common across species, followed by orthologous gene conversion utilizing the BioMart database. This approach yielded 11,923 common genes (Supplementary Table S2), which were then analyzed for expression levels and percentage of expression. Correlation analyses revealed that monkeys (*r* = 0.777, Mantel's test) demonstrated the closest transcriptomic similarities to humans, followed by mice (*r* = 0.752, Mantel's test), at the transcriptome level across all 18 common cell types (Fig. 1D). Furthermore, assessments of 50 hallmark gene sets from the MsigDB on the down-sampled data were consistent with these findings, with monkeys (*r* = 0.890, Spearman's correlation test) and mice (*r* = 0.865, Spearman's correlation test) showing the highest correlation with hallmark biological processes in humans (Fig. 1E).

### 3.2 Transcriptional similarities at the cell-type level

**Fig. 2:**
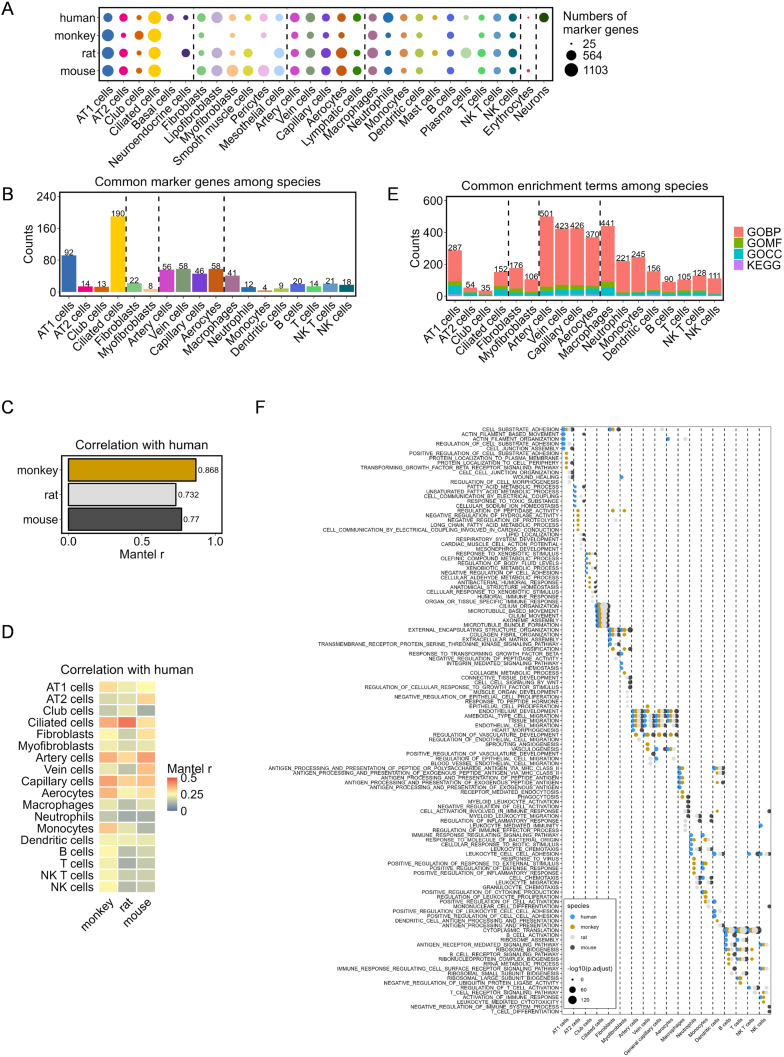
**Cross-species comparison of healthy lung cells at the cell type level. (A)** Number of marker genes of various lung cell types across different species. **(B)** Number of common marker genes in various lung cell types across four species. **(C)** Mantel's correlation analysis compares the expression level and percentage of common marker genes in randomly sampled lung cells of the human dataset with the other three. **(D)** Mantel's correlation analysis for comparing the expression and percentage of common marker genes in lung cell types of the human dataset with those in the other three. **(E)** Number of common enrichment GO and KEGG terms for different lung cell types across four species. **(F)** Top five common enrichment GOBP terms ordered according to the P-values in various cell types across five species.

Further analyses focused on cell-type-specific features by quantifying the expression of cell-type marker genes for each cell type across the four datasets (Supplementary Table S3, Fig. 2A). Variability in the number of cell-type marker genes was observed among species, with erythrocytes exhibiting the lowest and ciliated cells exhibiting the highest number of these genes. This highlights the specialized transcriptional landscape of ciliated cells. The specialization of ciliated cells was further demonstrated by their distinct clustering in the UMAP plots (Fig. 1A), affirming their unique transcriptional profiles.

Orthologous conversion of cell-type marker genes across species to their human equivalents revealed that ciliated cells possessed the highest number of common markers among the 18 cell types evaluated (Fig. 2B–Supplementary Table S4). To further characterize orthologous gene expression patterns across species, the top five common cell-type marker genes were identified from the fold changes for each cell type (Fig. S1). This analysis indicated that monkeys showed more marker genes overlapping with humans. Moreover, species-specific cell-type marker genes were cataloged (Supplementary Table S5), and the top five species-specific cell-type marker genes, determined from fold change, were presented (Fig. S2B). The minimum number of species-specific cell-type marker genes was 23, which was higher than the number of common cell-type marker genes in most cells. These markers were primarily species-specific (Fig. S2A). Given the unique cell-type marker gene numbers, this catalog showed that mice closely resembled humans in terms of lung epithelial cell types (Fig. S2A). Additionally, human neutrophils were characterized by the largest distinct set of cell-type marker genes, indicating a unique gene expression pattern that was absent in other species.

A comparative analysis of these genes within the down-sampled datasets revealed that monkeys (*r* = 0.833, Mantel's test) and mice (*r* = 0.767, Mantel's test) most closely mirrored those of humans across 11,923 common genes (Fig. 2C). Monkeys specifically demonstrated similar expression profiles to humans in epithelial, endothelial (excluding vein cells), and leukocyte clusters, whereas mice showed alignment with expression in human in mesenchymal and vein cell clusters (Fig. 2D).

Enrichment analysis across the 18 common cell types revealed differences in the frequency of shared enrichment terms (Supplementary Table S6). Despite the abundance of cell-type marker genes in ciliated cells, a corresponding prevalence of common enrichment terms was not observed, suggesting a high degree of specialization with limited but crucial biological functions. In contrast, macrophages and endothelial cells, which were widespread across various tissues, were associated with a diverse array of signaling pathways (Fig. 2E). Within the gene ontology biological process category, the analysis focused on the top five terms, with adjusted p-values for each cell type and species. This analysis indicated that while enrichment terms varied by cell type, fundamental biological processes remained conserved across species (Fig. 2F).

### 3.3 Conserved cell-type-specific TFs

**Fig. 3:**
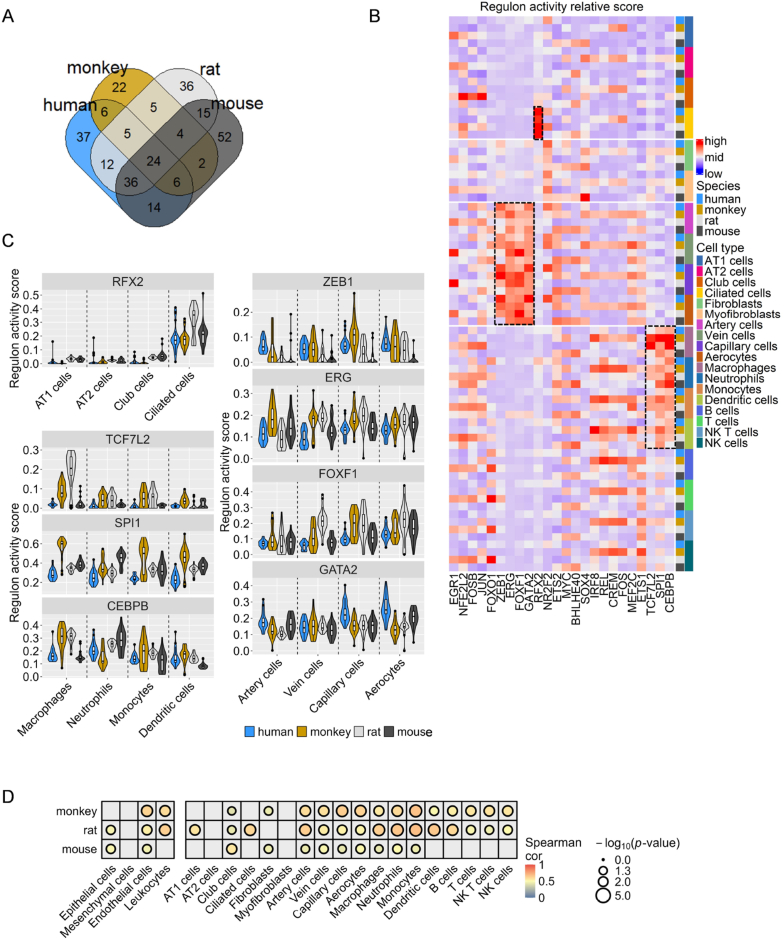
**Common TFs in healthy lung cells across species. (A)** Venn plot showing TFs across species. **(B)** Regulon activity for 24 common TFs across 18 common lung cell types of four species. The column is grouped into four main cell clusters, and TFs in rows are unsupervised clustered. Nine regulons from the three most active modules are highlighted with a dashed line. **(C)** Violin plots showing the activity distribution of each framed regulon in every cell type of various species. **(D)** Correlation analysis of the regulon activity of 24 common TFs in four main cell clusters (left) and 19 fine cell types (right) of lung cells. The analysis compares human data with those of other species; only *p* < 0.05 are shown.

Cell-type marker genes, which reflect cellular biological activities and processes, are frequently involved in complex regulatory networks of multiple genes. The SCENIC workflow enabled the identification and comparative analysis of critical TFs (Supplementary Table S7). This analysis revealed that mice possessed the highest number of TFs, with an unexpectedly large proportion—37.6 %, or 80 out of 213—shared with humans, exceeding the overlap between rats and mice. Conversely, monkeys demonstrated the fewest TFs, contributing an additional 36 common TFs among the other species (Fig. 3A).

Common TFs were conserved across species. The analysis detected 24 active TFs in all four species, which were segregated into three cell-type-specific groups with high activity through unsupervised clustering (Fig. 3B). Violin plots showed the activation scores of the eight most active TFs across species, displaying distinct activity patterns among various cell types. The specific activation of regulatory factor X2 (RFX2) in ciliated cells, Spi-1 proto-oncogene (SPI1) and CCAAT enhancer binding protein beta (CEBPB) in immune cells, and ETS transcription factor ERG (ERG), forkhead box F1 (FOXF1), and GATA binding protein 2 (GATA2) in endothelial cells were identified as critical for their respective physiological and biological functions (Fig. 3C). Moreover, certain TFs, including interferon regulatory factor 8 (IRF8), REL proto-oncogene (REL), cAMP responsive element modulator (CREM), and Fos proto-oncogene (FOS), showed increased activity in monkey lung cells, with similar species-specific activities observed in other species (Fig. 3B).

Correlation analysis was employed to evaluate the activity of 24 common TFs across the main and fine cell types in lung cells from human and nonhuman species (Fig. 3D). The analysis of the primary cell clusters revealed a significant correlation in endothelial cell regulator activities between nonhuman species and humans, particularly between humans and monkeys (*r* = 0.62, *p* < 0.01, Spearman's correlation test). However, no significant correlation was observed between human data and the mesenchymal cells of nonhuman species, the epithelial cells of monkeys, or the immune cells of mice. In fine cell types, club cell regulator activities in nonhuman species were correlated with those in humans, with the highest correlation observed in mice (*r* = 0.56, *p* < 0.01, Spearman's correlation test). Additionally, Alveolar type I (AT1) cells in rats and ciliated cells also showed significant similarities with human TF regulatory activity (*r* = 0.57, *p* < 0.01; *r* = 0.52, *p* < 0.01; Spearman's correlation test).

The presence of common TFs across species probably accounts for the similarities in biological functions among analogous cells, whereas species-specific TFs highlight the diversity in the functional capacities of more differentiated cell types. Unsupervised clustering of species-specific TFs identified four to five activation groups per species, aligning with broader cell population classifications. This clustering also revealed significantly high activation of TFs in specific cell types, for example, activation of nuclear factor I X (NFIX) and atonal bHLH transcription factor 8 (ATOH8) in human AT1 cells, MDS1 and EVI1 complex locus (MECOM) in Alveolar type II (AT2) cells, and ceramide synthase 4 (CERS4) in ciliated cells (Fig. S3).

### 3.4 Species-specific differences between sex and the lung transcriptome

**Fig. 4:**
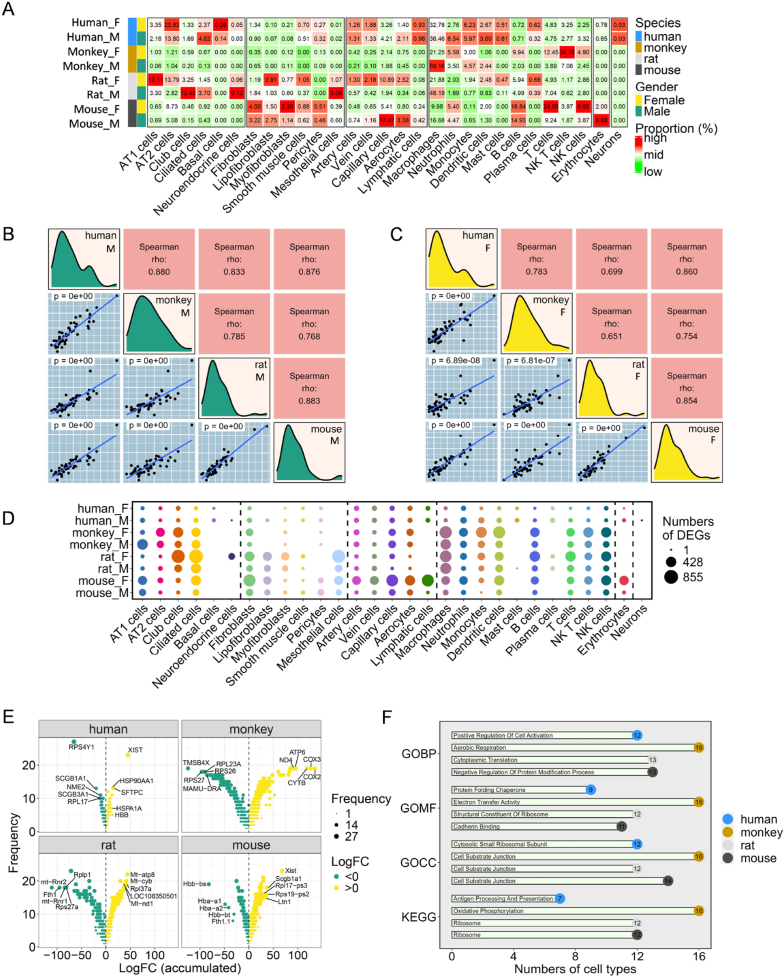
**Sex differences in healthy lung cells across species. (A)** Proportions of 29 fine cell typesclassified by sex across different species. **(B**–**C)** Correlation analysis of randomly sampled lung cells across species in various sex groups using module scores from 50 hallmark gene sets in the MsigDB dataset. **(D)** Sex-based DEGs across 29 fine celltypes. **(E)** Top five sex-based DEGs across species. The vertical axis of the volcano plot represents the number of occurrences across the 29 cell types, and the horizontal axis shows the accumulated LogFC in significant cell types. Label the top five genes on accumulated LogFC. **(F)** Top common enrichment terms for sex-based DEGs16 cell types across species.

In many vertebrates, sex differences represent the most significant phenotypic disparity among species. When categorizing lung cells according to sex in a steady state (Fig. S4A), the distribution of fine cell clusters was observed to remain consistent across sexes in humans. However, significant variations were observed in macrophage distribution across other species, particularly in lymphocytes in monkeys and mice and in capillary and epithelial cells in rats and mice (Fig. 4A). Analysis of sex ratios in fine cell types showed that rat samples aligned most closely with human patterns (*r* = 0.89, Mantel's test). However, mouse samples demonstrated the greatest deviations (*r* = 0.34, Mantel's test), which were largely attributed to differences in sex ratios of the sample size (Fig. S4B).

To assess the similarity of primary biological functions, down-sampled data from each species were compared using 50 hallmark gene sets from MsigDB, followed by a correlation analysis of the results. This analysis showed that, among males, monkeys (*r* = 0.880, Spearman's correlation test) and mice (*r* = 0.876, Spearman's correlation test) demonstrated the closest similarity to human males with respect to landmark biological processes (Fig. 4B). However, among females, mice (*r* = 0.860, Spearman's correlation test) and monkeys (*r* = 0.783, Spearman's correlation test) most closely mirrored human females (Fig. 4C).

Further investigation of sex-based DEGs revealed a limited number of sex-based DEGs across human lung fine cell types. Conversely, abundant sex-based DEGs were primarily observed in the immune cells, club cells, ciliated cells, and fibroblasts of other species (Fig. 4D–Supplementary Table S8). To show the distribution of sex-based DEGs at the fine cell type level for each species, the results were visualized using a volcano plot. In this plot, the x-axis represented the sum of log-fold change (logFC) across the 29 fine cell types, whereas the y-axis showed the number of clusters with significant differences. The top five DEGs with the largest sum of logFC are highlighted on the plot (Fig. 4E). Only 16 fine cell types across the four species exhibited at least five upregulated and five downregulated genes, with the results presented in standard volcano plots (Fig. S5). In human males, the most significant DEG was observed for ribosomal protein S4 Y-linked 1 (*RPS4Y1*), whereas in human and mouse females, it was observed for X inactive specific transcript (*XIST*). This pattern was not observed in other species. Additionally, in humans, the surfactant protein C (*SFTPC*), secretoglobin family 1A1 (*SCGB1A1*), and secretoglobin family 3A1 (*SCGB3A1*) showed sex-specific expression patterns. In mice, *SCGB1A1* was predominantly expressed in females, whereas in monkeys and rats, females tended to express mitochondrial genes. This suggests that sex plays a significant role in monkey and rat disease models influencing energy metabolism.

Based on these observations, GO and KEGG enrichment analyses were performed to identify the most variably enriched pathways across the cell type levels of each species (Fig. 4F–Supplementary Table S9). Monkeys demonstrated consistent enrichment across 16 cell types, primarily in pathways related to aerobic respiration, which highlighted that sex-based DEGs in monkeys primarily influence energy metabolism and consequently supporting the initial hypothesis. Conversely, in other species, particularly rats, sex-based DEGs were predominantly associated with ribosomal pathways, protein translation, and synthesis. Enrichment analysis with the lowest q-values for each species was also displayed, showing a significant enrichment in energy metabolism pathways among monkeys (Fig. S4). These findings emphasize the connection between sex and energy metabolism in monkeys. Additionally, given that genes with different sex expressions across species are primarily derived from club cells, ciliated cells, fibroblasts, and immune cells and that lung epithelial cells play a role in immunity, along with immune cells as the first mucosal barrier, the influence of sex on airway diseases should be acknowledged.

### 3.5 Similarities of airway cell response in animal models with that in human asthma

**Fig. 5:**
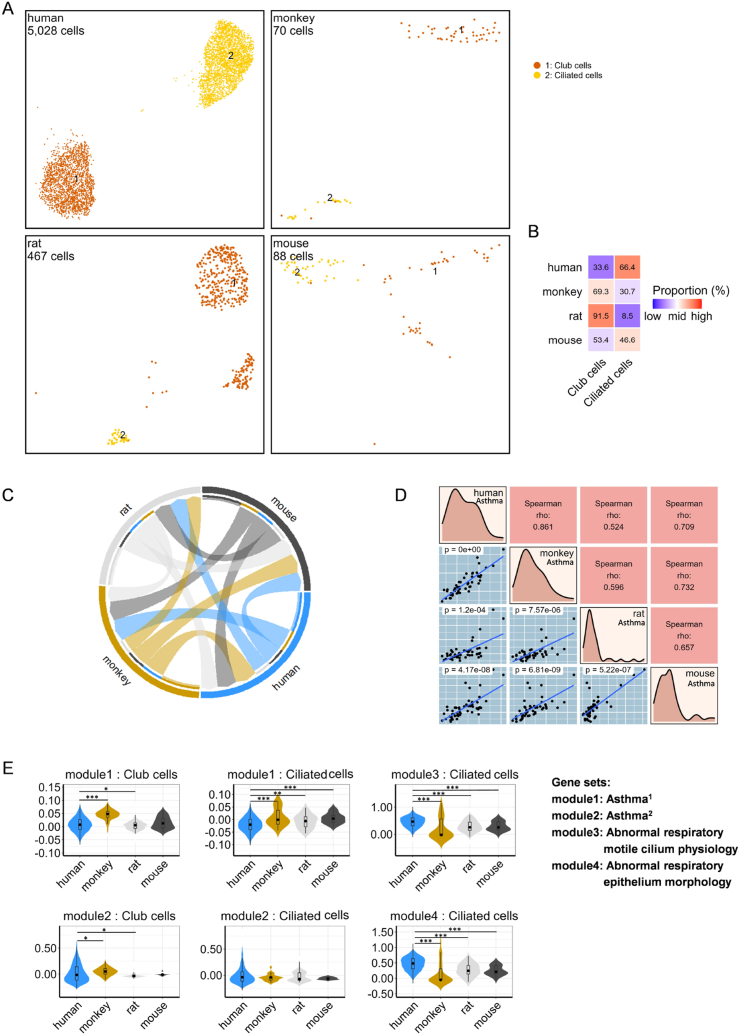
**Landscape of asthma lung cells and cross-species full-transcriptome comparison. (A)** UMAP plots show two fine cell types in the lungs of humans, monkeys, rats, and mice, respectively. Each cell type is color-coded and numbered, with the total number of cells in each data set shown in the top-left corner. **(B)** Proportions of the two fine cell types in different species. **(C)** Chord plot showing the correlation of full-transcriptome expression level and percentage in randomly sampled lung cells across species. Arrow width denotes the correlation coefficient from Mantel's test between species connected by a chord. **(D)** Correlation analysis of randomly sampled lung cells across species based on module scores from 50 hallmark gene sets from the MsigDB dataset. **(E)** Comparative graph of asthma and ciliated cells gene sets scores across species, all nonhuman species were compared with human, highlighting variations in the expression of genes related to asthma-specific biological processes (∗*p* < 0.05, ∗∗*p* < 0.01, ∗∗∗*p* < 0.001).

Asthmatic lung samples from four species were exclusively obtained from male participants. Human samples were obtained via bronchoscopic biopsy of the lower airways [34], and nonhuman species samples were derived from different models. These included the monkey *Ascaris suum* allergen model [35] and the mouse triple-allergen (DRA) model, which includes extracts of dust mite, ragweed, and *Aspergillus fumigatus* [36]. Although the rat ovalbumin (OVA) model only induced allergic airway inflammation [37], we retained it for analysis due to the limited availability of samples. Following rigorous quality control and data integration, cellular annotation was performed for each species. Owing to the constraints associated with specific sampling locales for human specimens, only club and ciliated cells were selected, resulting in a total of 5653 cells (Fig. 5A and B). After biomarker gene conversion, a corpus of 9786 cross-species common genes was identified (Supplementary Table S10).

**Fig. 6:**
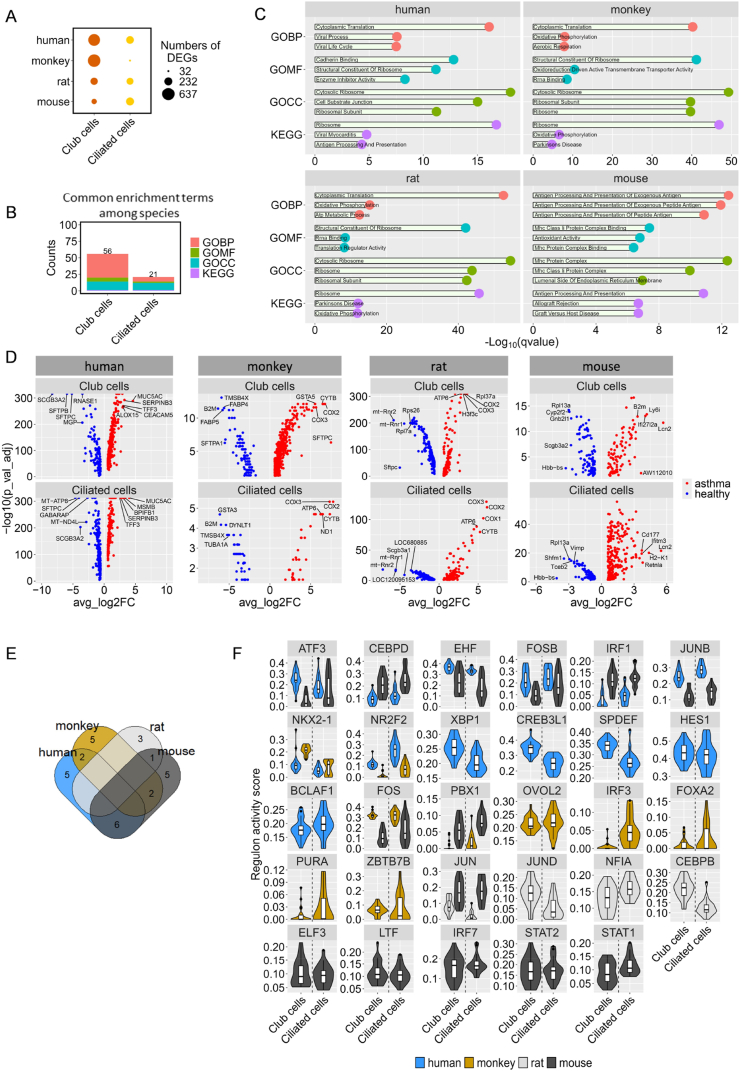
**Cross-species comparison of DEGs. (A)** Number of DEGs across various lung cell types in different species. **(B)** Number of common enrichment GO and KEGG terms in different lung cell types across four species. **(C)** Top three enrichment terms for common DEGs across species. These common genes were filtered from sex-basedDEGs, appearing in at least one of 16 cell types in every species. **(D)** DEGs in two common cell types across four species. **(E)** Venn plot showing TFs across species. **(F)** Violin plots showing the distribution of regulon activity in each cell type across different species.

Cross-species transcriptomic evaluations of the two distinct epithelial cell types showed that monkeys (*r* = 0.548, Mantel's test) demonstrated the highest similarity to human profiles, followed closely by mice (*r* = 0.480, Mantel's test) (Fig. 5C). Additionally, the 50 hallmark gene sets from MsigDB were utilized to score two distinct epithelial cell types across species, showing that monkeys (*r* = 0.861, Spearman's correlation test) and mice (*r* = 0.709, Spearman's correlation test) closely mirrored human data regarding key biological processes (Fig. 5D). Further analysis of asthma-relevant gene sets using violin plots demonstrated significant differences between monkey data and human models, with rat and mouse data aligned more closely with human profiles (Fig. 5E). Existing research highlights that ciliary dysfunction and ultrastructural abnormalities are features of patients with severe asthma [38], which led us to conduct additional assessments of gene sets for ciliary morphology, and physiology. Despite the differences between humans and other species, comparative analyses revealed that the genetic profiles of monkey-ciliated cells significantly diverge from those of their human counterparts. In contrast, rat and mouse-ciliated cells demonstrated greater alignment with human cellular functions (Fig. 5E). By integrating data from male lungs and asthma samples across species and focusing exclusively on club and ciliated cells, DEGs between homeostasis and asthma conditions were identified (Fig. 6A, Supplementary Table S11). Volcano plots showed that in monkeys and rats, asthma-related DEGs were predominantly related to mitochondrial functions (Fig. 6D). GO and KEGG analyses of these genes revealed a lack of common enrichment pathways in ciliated cells, highlighting species-specific variability (Fig. 6B–Supplementary Table S12). Human asthma-related DEGs were primarily associated with pathways regulating protein synthesis and degradation, whereas in monkeys and rats, they showed enrichment in pathways related to aerobic respiration and oxidative phosphorylation. Conversely, mouse genes showed predominant enrichment in pathways related to CD4 T cell-related immune responses (Fig. 6C).

TF analysis of asthma-related DEGs revealed six co-activated TFs between humans and mice, two between humans and monkeys, and none between humans and rats (Fig. 6E). The activation profiles of these TFs, particularly their increased presence under asthmatic conditions, highlighted the nuanced regulatory dynamics underlying the disease pathology (Fig. 6F). Among these, FosB proto-oncogene (FOSB) activation was observed across various cell types in healthy human patients, whereas ETS homologous factor (EHF) demonstrated specific activation in healthy monkeys. Additionally, SAM pointed domain containing ETS transcription factor (SPDEF) demonstrated increased activity in human club cells, highlighting the complex transcriptional landscape of asthma.

## 4 Discussion

In this study, we employed scRNA-seq to investigate the airway epithelium of humans, monkeys, mice, and rats, aiming to identify conserved and unique transcriptomic signatures. Additionally, we examined the influence of sex on gene expression, which remains unexplored in cross-species contexts. Our methodology integrated rigorous data processing, orthologous gene conversion, and advanced statistical analyses to ensure robust cross-species comparisons. By analyzing healthy and asthmatic lung samples, we sought to explain the mechanisms underlying species-specific disease manifestations and responses to asthma. These insights are crucial for the development of improved animal models.

This study provides a detailed transcriptomic landscape of the lungs across four species, comparing common cell types. Human samples exhibited the most diverse cell populations, while monkey samples exhibited fewer, illustrating the importance of sample size in identifying rare cell types, as observed by Jindal A [39]. Consistently, leukocytes were predominat in the lung cell populations in all species, a feature common to other human parenchymal organs [40]. However, this prevalence complicates single-cell analyses in immune-activated disease models, such as asthma; herein, where our samples contained fewer epithelial cells due to the absence of flow sorting and pre-processing.

In the comparison of whole transcriptome gene expression and marker gene set similarity scores across 18 common cell types, monkeys showed the closest resemblance to humans, which supports the evolutionary theory that non-human primates share the most similarities with humans [41]. Interestingly, our findings suggest that, contrary to the traditional view, mice are closer to humans than rats at the single-cell transcriptome level. This is consistent with previous multi-organ transcriptome studies, which demonstrated higher correlation coefficients between humans and mice than between humans and rats [42], and genomic studies that reported a higher rate of neutral DNA base substitutions in rats than in humans and mice [43].

At the cellular level, marker genes and TFs showed notable conservation. Previous studies have indicated that cell type-specific genes are highly conserved in human and mouse brain tissues, as revealed by single-cell analyses [44]. Our study revealed that common lung cell type marker genes had higher expression level correlations across species than did general genes, suggesting that these markers are essential for cross-species conservation. Both endothelial and immune cells displayed a significant number of shared enriched pathways, indicating that unique cell type marker genes also contribute to similar biological processes across species. Our research on conserved cell type-specific TFs, such as RFX2 [45,46], SPI1 [47], and ERG [48], provided insights into the conservations that support lung cell functions. These TFs were specifically activated in ciliated cells, myeloid cells, and endothelial cells, respectively, highlighting the cross-species conservation of TFs in regulating cell-specific functions and responses.

Sex differences in disease incidence and mortality have been well-documented across various respiratory conditions, including higher rates in male patients with coronavirus disease 2019 and interstitial lung diseases [49,50] and increased susceptibility to allergic conditions in females [51]. Our cross-species and sex-specific comparisons under healthy conditions revealed higher proportions of NK and T cells in female mice and monkeys. This observation is consistent with previous findings, which suggested that female mice exhibit more efficient acute inflammatory responses [52]. However, other studies suggest that the protective effect in female mice against severe acute respiratory syndrome coronavirus infection is associated with estrogen receptor signaling rather than with T and B cell responses [53]. T cell immunity is closely associated with cancer. The choice of first-line treatment for non-small cell lung cancer patients with high PD-L1 expression remains controversial [54]. Additionally, sex differences in efficacy have been observed in these patients when treated with anti-PD-1 monotherapy targeting the T cell receptor [55]. However, our study found no significant sex differences in the proportion of T cells in human lungs. This suggests that sex-based differences in immunity might not be solely determined by T cell counts but could involve more complex biological mechanisms. Further evidence is needed to clarify whether immune checkpoint inhibitors (ICIs) produce adverse effects through T cells [56,57].

Conversely, female humans and mice exhibited high levels of *XIST/Xist* RNA expression in their lungs, whereas female monkeys and rats did not, despite previous reports indicating *XIST/Xist* RNA expression in these species [58,59]. *XIST/Xist* initiates the X chromosome inactivation (XCI) process by depositing heterochromatic histone modifications that silence the alleles [60]. Previous studies revealed the absence of *XIST/Xist* RNA “cloud” in mature lymphocytes and AT2 cells in female mice and humans, leading to X-linked gene escape from XCI and significant sex-biased gene expression [[61], [62], [63]]. Recent research indicates that *Xist* ribonucleoproteins promote female-biased autoimmunity [64]. Furthermore, in this study, we revealed that sex-based DEGs in monkeys and rats, which lack *XIST* expression, were significantly enriched in aerobic respiration and oxidative phosphorylation pathways. Conversely, this enrichment was not observed in humans and mice expressing *XIST*. Therefore, we speculate that *XIST* plays a role in suppressing the sex-based DEGs in the lungs. The upregulation of oxidative phosphorylation in human skeletal muscle cells and myotubes is associated with female *XIST* expression [65], which in inconsistent with our findings in the lungs. This discrepancy may be attributed to the distinct biological functions of various organs. However, further research is needed to validate our hypotheses.

Given the abundance of sex-related DEGs in club and ciliated cells, we focused on epithelial cells in asthma to evaluate cross-species responses in airway cells. Abnormalities in these cells lead to impaired airway mucus clearance in asthma, particularly owing to ciliated cell damage and MUC5AC^+^ mucus plug formation [38,66]. The monkey *Ascaris suum* allergen model and the mouse DRA model in our study showed the highest transcriptional similarity to human asthma. The mouse DRA model also shared the most predicted TFs from asthma-related DEGs, indicating their relevance in simulating human asthma. However, the monkey *Ascaris suum* allergen model did not express genes related to abnormal ciliary morphology, function, and mucociliary differentiation. Enrichment analysis revealed that DEGs in monkeys and rats were significantly enriched in aerobic respiration and oxidative phosphorylation pathways. These findings highlight the limitations of the monkey *Ascaris suum* allergen and rat OVA models in simulating patients with asthma. Inflammation is a fundamental hallmark of cancer, significantly influencing tumorigenesis and tumor progression. Elevated levels of lactate dehydrogenase (LDH), which are indicative of altered energy metabolism in human lung tumors, are associated with poorer clinical outcomes [67]. This relationship may explain why human asthma, an inflammatory disease, is not associated with enhanced activity of the oxidative phosphorylation pathway. Additionally, muc5ac is highly expressed in the epithelial cells of human patients with asthma, where mucus cell hyperplasia, originating from a novel mucociliary cell state, and goblet cell hyperplasia have been observed [34]. However, this hyperplasia was not observed in animal models. Enhancing the fidelity of animal models of human asthma is pivotal for developing more effective and personalized therapeutic strategies.

Our study lays the groundwork for such advancements by identifying key strengths and limitations of current models, thereby guiding future research in respiratory disease.

## 5 Limitation

This study offers numerous novel insights; however, it also has certain limitations, including uneven sample sizes and inherent constraints of the single-cell sequencing technology. Hence, future research should aim to address these challenges by increasing sample sizes, employing multimodal analysis methods, and advancing the sequencing techniques.

## 6 Conclusion

Our study offers a comprehensive cross-species and sex-specific analysis of the airway epithelium utilizing scRNA-seq. Crucial transcriptomic similarities and differences among humans, monkeys, mice, and rats were identified, providing valuable insights into species-specific disease manifestations and responses to asthma. Our findings highlight the influence of sex on gene expression, highlighting its potential effect on disease susceptibility and progression. The identification of conserved and unique cellular markers and TFs across species elucidates respiratory disease mechanisms and informs the refinement of animal models for better translation to human conditions. These insights will pave the way for optimizing animal models to more accurately reflect human respiratory diseases. Further research should focus on expanding sample sizes, incorporating multimodal analysis techniques, and advancing the sequencing technologies to address current limitations and offer deeper insights into the cellular dynamics of respiratory diseases.

## Data availability

Public data can be found through GEO accession numbers: healthy human datasets were from GSE122960, GSE128033, GSE133747, GSE135851, GSE135893, GSE136831, GSE159354, GSE168191, GSE173896. Healthy mouse datasets were from oudlGSE196794 and China National GeneBank DataBase IDs CNP0002884. Healthy rat datasets were from GSE133747, GSE252844 and GSE211925. Healthy mouse datasets were from GSE129605, GSE133747, GSE133992, GSE145435, GSE151374, GSE155436, GSE155814, GSE164621 and GSE185256. Asthma datasets of nonhuman species were obtained from GSE213085, and GSE203656. The asthma data of humans can be accessed at European Genome–phenome Archive: EGAS00001002649, and were kindly provided by FA Vieira Braga, who our teammate Zhaoyu Song connected with. We have also provided sample details (Supplementary Table S13).

## Code reproducibility

Codes are provided in supplementary materials.

## Ethics approval

Not applicable.

## Funding information

This research was supported by the 10.13039/501100001809National Natural Science Foundation of China (82100077 and 82070001), Tianjin Second Batch of Health Industry High-Level Talent Selection and Training Project (TJSQNYXXR-D2-070), 10.13039/501100006606Natural Science Foundation of Tianjin (23JCYBJC01560 and 21JCZDJC00430), Science and Technology Planning Project of the 10.13039/501100010882Tianjin Municipal Education Commission (2022YGYB14), and Tianjin Key Medical Discipline (Specialty) Construction Project (TJYXZDXK-067C and TJYXZDXK-063B).

## CRediT authorship contribution statement

**Biyu Gui:** Writing – original draft, Software, Project administration, Investigation, Data curation. **Qi Wang:** Writing – original draft, Software, Methodology, Formal analysis, Data curation. **Jianhai Wang:** Writing – review & editing, Data curation. **Xue Li:** Writing – review & editing. **Qi Wu:** Supervision, Conceptualization. **Huaiyong Chen:** Writing – review & editing, Supervision, Methodology.

## Declaration of competing interest

The authors declare that they have no known competing financial interests or personal relationships that could have appeared to influence the work reported in this paper.

## References

[1] Franks T.J., Colby T.V., Travis W.D. (2008). Resident cellular components of the human lung: current knowledge and goals for research on cell phenotyping and function. Proc. Am. Thorac. Soc..

[2] Sikkema L., Ramírez-Suástegui C., Strobl D.C. (2023). An integrated cell atlas of the lung in health and disease. Nat. Med..

[3] Pennitz P., Kirsten H., Friedrich V.D. (2022). A pulmonologist's guide to perform and analyse cross-species single lung cell transcriptomics. Eur. Respir. Rev. : an Official Journal of the European Respiratory Society.

[4] Chen D., Sun J., Zhu J. (2021). Single cell atlas for 11 non-model mammals, reptiles and birds. Nat. Commun..

[5] Travaglini K.J., Nabhan A.N., Penland L. (2020). A molecular cell atlas of the human lung from single-cell RNA sequencing. Nature.

[6] Muus C., Luecken M.D., Eraslan G. (2021). Single-cell meta-analysis of SARS-CoV-2 entry genes across tissues and demographics. Nat. Med..

[7] Tang M., Elicker B.M., Henry T. (2022). Mucus plugs persist in asthma, and changes in mucus plugs associate with changes in airflow over time. Am. J. Respir. Crit. Care Med..

[8] Koh K.D., Bonser L.R., Eckalbar W.L. (2023). Genomic characterization and therapeutic utilization of IL-13-responsive sequences in asthma. Cell Genomics.

[9] Seibold M.A. (2018). Interleukin-13 stimulation reveals the cellular and functional plasticity of the airway epithelium. Annals of the American Thoracic Society.

[10] Okuda K., Chen G., Subramani D.B. (2019). Localization of secretory mucins MUC5AC and MUC5B in normal/healthy human airways. Am. J. Respir. Crit. Care Med..

[11] Li K., Zhang Q., Li L. (2022). DJ-1 governs airway progenitor cell/eosinophil interactions to promote allergic inflammation. J. Allergy Clin. Immunol..

[12] R Core Team (2022). https://www.R-project.org/.

[13] Hao Y., Hao S., Andersen-Nissen E. (2021). Integrated analysis of multimodal single-cell data. Cell.

[14] Wickham H., Averick M., Bryan J. (2019). Welcome to the tidyverse. J. Open Source Softw..

[15] McGinnis C.S., Murrow L.M., Gartner Z.J. (2019). DoubletFinder: doublet detection in single-cell RNA sequencing data using artificial nearest neighbors. Cell Systems.

[16] Bates D., Maechler M., Jagan M. (2024). Matrix: sparse and dense matrix classes and methods. https://CRAN.R-project.org/package=Matrix.

[17] Korsunsky I., Millard N., Fan J. (2019). Fast, sensitive and accurate integration of single-cell data with Harmony. Nat. Methods.

[18] Dolgalev I. (2022). Msigdbr: MSigDB gene sets for multiple organisms in a tidy data format. R package version 7.5.1).

[19] Wu T., Hu E., Xu S. (2021). clusterProfiler 4.0: a universal enrichment tool for interpreting omics data. Innovation.

[20] Carlson M. (2022). org. Hs.eg.db: Genome wide annotation for Human.

[21] Carlson M. (2022). org.Mmu.eg.db: Genome wide annotation for Rhesus.

[22] Carlson M. (2022). org.Mm.eg.db: Genome wide annotation for Mouse.

[23] Carlson M. (2022). org.Rn.eg.db: Genome wide annotation for Rat.

[24] Aibar S., González-Blas C.B., Moerman T. (2017). SCENIC: single-cell regulatory network inference and clustering. Nat. Methods.

[25] Huynh-Thu V.A., Irrthum A., Wehenkel L. (2010). Inferring regulatory networks from expression data using tree-based methods. PLoS One.

[26] Bougeard S., Dray S. (2018). Supervised multiblock analysis in R with the ade4 package. J. Stat. Software.

[27] Gu Z., Gu L., Eils R. (2014). Circlize Implements and enhances circular visualization in R. Bioinformatics.

[28] Gu Z., Eils R., Schlesner M. (2016). Complex heatmaps reveal patterns and correlations in multidimensional genomic data. Bioinformatics.

[29] Chen H. (2022). VennDiagram: generate high-resolution Venn and euler plots (R package version 1.7.3). https://CRAN.R-project.org/package=VennDiagram.

[30] Villanueva R.A.M., Chen Z.J. (2019). ggplot2: elegant graphics for data analysis. Measurement: Interdisciplinary Research and Perspectives.

[31] Schloerke B., Cook D., Larmarange J. (2024). GGally: extension to'ggplot2' (R package version 2.2.1). https://CRAN.R-project.org/package=GGally.

[32] Wilke C.O., Wickham H., Wilke M.C.O. (2024). Cowplot: streamlined plot theme and plot annotations for 'ggplot2' (R package version 1.1.3). https://CRAN.R-project.org/package=cowplot.

[33] Kassambara A. (2023). Ggpubr: 'ggplot2' based publication ready plots. R package version 0.6.0).

[34] Vieira Braga F.A., Kar G., Berg M. (2019). A cellular census of human lungs identifies novel cell states in health and in asthma. Nat. Med..

[35] Wang Y., Dong X., Pan C. (2022). Single-cell transcriptomic characterization reveals the landscape of airway remodeling and inflammation in a cynomolgus monkey model of asthma. Front. Immunol..

[36] Kim J.Y., Stevens P., Karpurapu M. (2022). Targeting ETosis by miR-155 inhibition mitigates mixed granulocytic asthmatic lung inflammation. Front. Immunol..

[37] Chu M., Gao H., Esparza P. (2023). Chronic developmental hypoxia alters rat lung immune cell transcriptomes during allergic airway inflammation. Physiological Reports.

[38] Thomas B., Rutman A., Hirst R.A. (2010). Ciliary dysfunction and ultrastructural abnormalities are features of severe asthma. J. Allergy Clin. Immunol..

[39] Jindal A., Gupta P., Jayadeva (2018). Discovery of rare cells from voluminous single cell expression data. Nat. Commun..

[40] He S., Wang L.-H., Liu Y. (2020). Single-cell transcriptome profiling of an adult human cell atlas of 15 major organs. Genome Biol..

[41] Kuderna L.F.K., Ulirsch J.C., Rashid S. (2024). Identification of constrained sequence elements across 239 primate genomes. Nature.

[42] Prasad A., Kumar S.S., Dessimoz C. (2013). Global regulatory architecture of human, mouse and rat tissue transcriptomes. BMC Genom..

[43] Gibbs R.A., Weinstock G.M., Metzker M.L. (2004). Genome sequence of the Brown Norway rat yields insights into mammalian evolution. Nature.

[44] La Manno G., Gyllborg D., Codeluppi S. (2016). Molecular diversity of midbrain development in mouse, human, and stem cells. Cell.

[45] Coyle M.C., Tajima A.M., Leon F. (2023). An RFX transcription factor regulates ciliogenesis in the closest living relatives of animals. Curr. Biol. : CB.

[46] Lemeille S., Paschaki M., Baas D. (2020). Interplay of RFX transcription factors 1, 2 and 3 in motile ciliogenesis. Nucleic Acids Res..

[47] Zakrzewska A., Cui C., Stockhammer O.W. (2010). Macrophage-specific gene functions in Spi1-directed innate immunity. Blood.

[48] Caporarello N., Lee J., Pham T.X. (2022). Dysfunctional ERG signaling drives pulmonary vascular aging and persistent fibrosis. Nat. Commun..

[49] Mwananyanda L., Gill C.J., MacLeod W. (2021). Covid-19 deaths in Africa: prospective systematic postmortem surveillance study. BMJ (Clinical Research ed).

[50] Kawano-Dourado L., Glassberg M.K., Assayag D. (2021). Sex and gender in interstitial lung diseases. Eur. Respir. Rev. : an Official Journal of the European Respiratory Society.

[51] Shin Y.H., Hwang J., Kwon R. (2023). Global, regional, and national burden of allergic disorders and their risk factors in 204 countries and territories, from 1990 to 2019: a systematic analysis for the Global Burden of Disease Study 2019. Allergy.

[52] Scotland R.S., Stables M.J., Madalli S. (2011). Sex differences in resident immune cell phenotype underlie more efficient acute inflammatory responses in female mice. Blood.

[53] Channappanavar R., Fett C., Mack M. (2017). Sex-based differences in susceptibility to severe acute respiratory syndrome coronavirus infection. J. Immunol..

[54] Rizzo A. (2022). Identifying optimal first-line treatment for advanced non-small cell lung carcinoma with high PD-L1 expression: a matter of debate. Br. J. Cancer.

[55] Conforti F., Pala L., Pagan E. (2021). Sex-based differences in response to anti-PD-1 or PD-L1 treatment in patients with non-small-cell lung cancer expressing high PD-L1 levels. A systematic review and meta-analysis of randomized clinical trials. ESMO Open.

[56] Rizzo A., Santoni M., Mollica V. (2021). Peripheral neuropathy and headache in cancer patients treated with immunotherapy and immuno-oncology combinations: the MOUSEION-02 study. Expet Opin. Drug Metabol. Toxicol..

[57] Guven D.C., Erul E., Kaygusuz Y. (2023). Immune checkpoint inhibitor-related hearing loss: a systematic review and analysis of individual patient data. Support. Care Cancer : Official Journal of the Multinational Association of Supportive Care In Cancer.

[58] Reinius B., Saetre P., Leonard J.A. (2008). An evolutionarily conserved sexual signature in the primate brain. PLoS Genet..

[59] Liang K., Cui M., Fu X. (2021). LncRNA Xist induces arterial smooth muscle cell apoptosis in thoracic aortic aneurysm through miR-29b-3p/Eln pathway. Biomed. Pharmacother..

[60] Brockdorff N., Bowness J.S., Wei G. (2020). Progress toward understanding chromosome silencing by Xist RNA. Gene Dev..

[61] Sierra I., Pyfrom S., Weiner A. (2023). Unusual X chromosome inactivation maintenance in female alveolar type 2 cells is correlated with increased numbers of X-linked escape genes and sex-biased gene expression. Stem Cell Rep..

[62] Syrett C.M., Sindhava V., Hodawadekar S. (2017). Loss of Xist RNA from the inactive X during B cell development is restored in a dynamic YY1-dependent two-step process in activated B cells. PLoS Genet..

[63] Syrett C.M., Paneru B., Sandoval-Heglund D. (2019). Altered X-chromosome inactivation in T cells may promote sex-biased autoimmune diseases. JCI Insight.

[64] Dou D.R., Zhao Y., Belk J.A. (2024). Xist ribonucleoproteins promote female sex-biased autoimmunity. Cell.

[65] Davegårdh C., Hall Wedin E., Broholm C. (2019). Sex influences DNA methylation and gene expression in human skeletal muscle myoblasts and myotubes. Stem Cell Res. Ther..

[66] Bonser L.R., Zlock L., Finkbeiner W. (2016). Epithelial tethering of MUC5AC-rich mucus impairs mucociliary transport in asthma. J. Clin. Investig..

[67] Sahin T.K., Rizzo A., Aksoy S. (2024). Prognostic significance of the royal marsden hospital (RMH) score in patients with cancer: a systematic review and meta-analysis. Cancers.

