# Supplementary material for: Cross-species comparison of airway epithelium transcriptomics
Source: Heliyon. 2024 Sep 22;10(19):e38259. doi: 10.1016/j.heliyon.2024.e38259 (PMC11466595; doi:10.1016/j.heliyon.2024.e38259)
Supplement: Multimedia component 15 [file mmc15.docx]

**Figure S1. Top 5 common marker genes for each healthy lung cell type across five species.**

**Figure S2. Species-specific marker genes in 18 common lung cell types.**

**(A)** Unique marker gene counts in various lung cell types. **(B)** Top five unique marker genes of each lung cell type across four species.

**Figure S3. Species-specific TFs across healthy lung cell types.**

Regulon activity of unique TFs in 18 common lung cell types of human **(A)**, monkey **(B)**, rat **(C)**, and mouse **(D)**. Columns are divided into four main cell clusters, and TFs in rows are unsupervised clustered within each species.

**Figure S4. Sample attributes and sex-based top enrichment terms across four species.**

**(A)** Sample size for healthy lung tissue, categorized by sex, across different species. **(B)** Mantel’s correlation analysis comparing the percentage of 29 fine cell typers in the human dataset with those in the other three species. **(C)** Top three enrichment terms for common sex-based DEGs across species. Common genes were filtered from the sex-based DEGs that appear in at least one of the 16 cell types in every species.

**Figure S5. Sex-based DEGs in 16 common cell types across four species.**
